# Bread Biopreservation through the Addition of Lactic Acid Bacteria in Sourdough

**DOI:** 10.3390/foods12040864

**Published:** 2023-02-17

**Authors:** Francisco Illueca, Ana Moreno, Jorge Calpe, Tiago de Melo Nazareth, Victor Dopazo, Giuseppe Meca, Juan Manuel Quiles, Carlos Luz

**Affiliations:** 1 Department of Food Science and Toxicology, Faculty of Pharmacy, University of Valencia, Ave. Vicent Andrés Estellés s/n, 46100 Burjassot, Spain; 2 AgrotechUV Incubator, Scientific Park of University of Valence, St. Catedrático Agustín Escardino 9, 46980 Paterna, Spain

**Keywords:** antifungal, phenolic acids, volatile organic compounds, organic acid, fungi

## Abstract

Nowadays, the consumer seeks to replace synthetic preservatives with biopreservation methods, such as sourdough in bread. Lactic acid bacteria (LAB) are used as starter cultures in many food products. In this work, commercial yeast bread and sourdough breads were prepared as controls, as well as sourdough breads with *L. plantarum* 5L1 lyophilized. The impact of *L. plantarum* 5L1 on the properties of bread was studied. Antifungal compounds and the impact on the protein fraction by the different treatments in doughs and breads were also analyzed. In addition, the biopreservation capacity of the treatments in breads contaminated with fungi was studied and the mycotoxin content was analyzed. The results showed significant differences with respect to the controls in the properties of the bread and a higher total phenolic and lactic acid content in breads with higher amounts of *L. plantarum* 5L1. In addition, there was a higher content of alcohol and esters. Furthermore, adding this starter culture produced hydrolysis of the 50 kDa band proteins. Finally, the higher concentration of *L. plantarum* 5L1 delayed fungal growth and reduced the content of AFB1 and AFB2 compared to the control.

## 1. Introduction

Bread is a perishable food, and bacteria, yeasts, and fungi can cause its deterioration. It has a high humidity percentage, a water activity (a_w_) between 0.94 and 0.97, and a pH close to 6. Therefore, storage temperatures between 25–30 °C cause a greater probability of contamination after baking since it is the optimum temperature range for fungal growth. This is the most critical phase due to storage, wrapping, or cutting conditions [1]. The main contaminating microorganisms are fungi, with the most common genera being *Aspergillus*, *Penicillium*, *Mucor*, *Monilia*, *Endomyces*, *Cladosporium*, *Fusarium*, and *Rhizopus* [2]. Different strategies can be used to destroy the spores to avoid food deterioration, including infrared radiation, ethanol, propionic acid, packaging techniques, or sourdough. The latter increases the shelf life due to reduced pH and lactic and acetic acid production by lactic acid bacteria (LAB) [3].

The growth of fungi, bacteria, or yeast produces tremendous economic losses at the industrial level and rejection by the consumer. Moreover, fungal contamination significantly impacts food’s nutritional and organoleptic characteristics, so fungi may affect the harvesting, processing, or consumption stage. This is mainly due to the large production of spores that are transmitted via the air. In addition, they can develop in unfavorable environments such as low temperatures, acidic or basic pH, low a_w_, and high concentrations of preservatives [4].

The genus *Aspergillus* can grow in a temperature range from 6 °C to 48 °C, with the optimum temperature being 25 °C. They can also develop between a pH of 2 and 11.2 or in a_w_ close to 0.85. Therefore, the main foods affected by this genus of fungus are cereals, fruit, nuts, and seeds. In addition, within this group, *Aspergillus flavus* or *Aspergillus parasiticus* are the largest producers of aflatoxins (AF), and in the case of *Aspergillus carbonarius,* it is the main producer of Ochratoxin A (OTA) [4]. The genus *Penicillium* can grow in a temperature range between −2 °C and 37 °C, with the optimum temperature being 25 °C, and in a pH range between 1.6 and 10. The optimum a_w_ for its growth is close to 0.8, and it can grow at low oxygen concentrations. On the other hand, the main toxins they can produce are OTA, produced by *Penicillium verrucosum*; citrin, produced by *Penicillium expansum or Penicillium citrinum*; and cyclopiazonic acid, produced by *Penicillium camemberti* or *Penicillium commune* [4].

Biopreservation uses the antimicrobial potential of the food microbiota itself or that of the metabolites produced by it to prolong the shelf life of food and maintain quality for as long as possible [5]. An example is the fermentation of sourdough, which produces microbial metabolites that contribute to the flavor, aroma, texture, digestibility, nutritional quality, and preservation of bread. Sourdough fermentation is a conservative effect exerted by LAB, mainly due to the production of antimicrobial metabolites such as organic acids and phenolic compounds [6].

LAB has three mechanisms that determine its antifungal action. The first of these is its ability to produce antimicrobial compounds. The action of lactic acid stands out, although it is also worth noting the effects of other compounds such as phenolic acids, hydrogen peroxide, short-chain organic acids, and bioactive peptides. The second is due to competition for nutrients by LAB, because they are highly integrated into the substrate in which they grow. The last mechanism consists of the pH reduction produced during fermentation, which creates challenging conditions for the development of the fungus [7].

In recent years, the use of LAB in food has increased. These are capable of producing antifungal compounds, especially *L. plantarum,* against fungi of the genus *Aspergillus.* Therefore, different strains of *L. plantarum* are used as sourdough starter cultures [2]. The use of starter cultures in preparing sourdough can lead to standardized products in which a reduction in biodiversity is observed, compared to spontaneous sourdough. These starter cultures must withstand drying and adapt to the cereal matrix. Thus, freeze-dried starter cultures that combine various LAB strains are being developed and are producing better results [8]. These starter cultures aim to reduce the fermentation time used in spontaneous sourdough. In addition, an improvement in the sensory attributes of bread is intended [9].

The aims of this study were: (a) to evaluate the impact of *L. plantarum* 5L1 on the properties of bread; (b) to identify antifungal compounds produced during sourdough fermentation; (c) to determine the impact of *L. plantarum* 5L1 on the protein fraction of doughs and breads; (d) to study the biopreservation capacity of sourdoughs with *L. plantarum* 5L1 against fungi of the genera *Aspergillus* and *Penicillium*; and € to analyze the reduction of the mycotoxin content by sourdoughs with *L. plantarum* 5L1 in breads contaminated with fungi of the genera *Aspergillus* and *Penicillium.*

Therefore, this study helps fill a literary gap since we can highlight the use of *L. plantarum* 5L1 as a biocontrol to improve the shelf life and reduce the possible production of mycotoxins by some fungal species.

## 2. Materials and Methods

### 2.1. Chemicals

Deionized water was obtained from the Milli-Q purification kit (Millipore Corp., Bedford, MA, USA). Solvents grade liquid chromatography was obtained from VWR Chemicals (Radnor, PA, USA), including acetonitrile, methanol, ethyl acetate (EA), formic acid (FA) and sulfuric acid (SA) (99%). Magnesium sulfate, C18, ammonium formate, sodium chloride, tween reagent, and glycerol were obtained from Sigma-Aldrich (St. Louis, MO, USA). Buffered peptone water was obtained from Liofilchem Bacteriology Products (Roseto, Italy). Sodium hydroxide (NaOH), sodium carbonate (NaCO_3_), and calcium propionate were purchased from Fisher Scientific (Loughborough, UK).

Lactic and acetic acids standards, Folin–Ciocalteu reagent, and “1,1-diphenyl-2-picryl-hydrazil” (DPPH) were provided by Sigma-Aldrich (St. Louis, MO, USA). Gallic acid was obtained from UCB (Brussels, Belgium). Man Rogosa Sharpe-Agar (MRS-A), Man Rogosa Sharpe-Broth (MRS-B), Potato Dextrose Agar (PDA), and Potato Dextrose Broth (PDB) culture media were obtained from Liofilchem (Teramo, Italy). Whey was purchased from Alclipor Company, S. A. L. (Benassal, Spain). Once collected, the pH was measured and was approximately 3.6, then the whey was frozen. Subsequently, it was thawed and pasteurized. This sweet goat whey was obtained by the action of coagulating enzymes on milk casein. This product has the following composition per 100 g: 93.1 g of water, 0.4 g of fat (0.2 g saturated, 0.1 g monounsaturated, <0.1 g polyunsaturated, and 2 mg of cholesterol), 5.1 g of carbohydrates (lactose), 0.9 g of protein, and <0.1 g of sodium.

Acetone and tris(hydroxymethyl)aminomethane were provided by Scharlab S.L. (Sentmenat, Spain). Sigma-Aldrich provided the sodium dodecyl sulfate (SDS), Trizma Buffer solution, and bromophenol blue (St. Louis, MO, USA). Glacial acetic acid and dithiothreitol (DDT) were purchased from Fisher Scientific (Loughborough, UK) and glycine was purchased from VWR Chemicals (Radnor, PA, USA).

### 2.2. Microorganisms and Culture Conditions

The LAB used in this work was *L. plantarum* 5L1. It was identified and deposited on the Spanish Collection of Type Cultures (CECT) at the University of Valencia (Valencia, Spain) and was preserved in sterile 30% glycerol and stored at −80 °C before use. Before the fermentation experiments, LAB was cultured in MRS-B at 37 °C for 48 h under anaerobic conditions.

The fungi used were *Aspergillus flavus* ISPA 8111 (*A. flavus)* from the Institute of Sciences of Food Production ISPA-CNR (Bari, Italy), *Penicillium verrucosum* VTT D 01847 (*P. verrucosum)* obtained from VTT Technical Research Center (Espoo, Finland), and *Penicillium commune* CECT 20767 (*P. commune)*. These microorganisms were maintained in sterile 30% glycerol at −80 °C. They were then recovered in PDB broth at 25 °C and inoculated in PDA Petri dishes. The spores were obtained at the time of analysis.

### 2.3. Sourdough Fermentation with LAB Lyophilized

LAB was cultivated in MRS-B at 37 °C until the exponential growth phase (10 h). Next, the content of the fermented medium was distributed in 50 mL tubes and centrifuged at 7700× *g*, 4 °C for 15 min. Afterward, the supernatant was discarded and the bacterial cells were resuspended in 5 mL of previously pasteurized 1/5 diluted whey. Finally, the content was stored at −80 °C and subsequently lyophilized. The concentration of this starter culture was 10^12^ CFU/g.

Three different sourdoughs of 200 g were prepared. The first was a control and was prepared in duplicate (SC). This only contained sterile mineral water and flour, giving rise to a spontaneously fermented sourdough. In the second (S_1_) and third (S_2_), 0.5% and 5% of starter culture was added, respectively, decreasing the flour content. In addition, a control bread without sourdough was also made. The mixtures of corresponding ingredients were made in sterile flasks and left stirring for 12 h at 37 °C. The final compositions of these sourdoughs are shown in Table 1.

### 2.4. Sourdough Bread Preparation

The sourdoughs were used to produce bread. The bread doughs were prepared by replacing 25% of the total weight of the ingredients in the form of sourdough compared to the control bread. In addition, 0.2% of calcium propionate was added to one of the doughs with spontaneous fermentation sourdough relative to the total weight of the bread (800 g). The two breads incorporating a starter culture sourdough theoretically had LAB concentrations of 1.25 × 10^9^ CFU/g and 1.25 × 10^10^ CFU/g, respectively. The final compositions of the wheat dough formulations are shown in Table 2.

**Figure 1:**
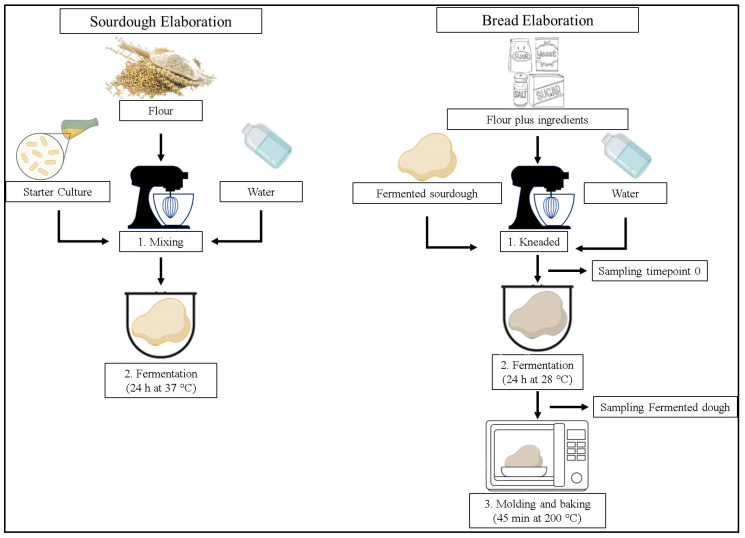
Bread production diagram.

The method described by Rosell et al. [10] was used for bread manufacture. A production diagram is shown in Figure 1. First, the warm water, sugar, and yeast were mixed in a beaker. The flour, salt, sourdough, and the mixture from step 1 were then added to the Cecomixer Easy mixer from Cecotec (Valencia, Spain), leaving it to knead for 5 min. The doughs were then fermented for 24 h at 28 °C, except for the control, which was only fermented for 1 h. Subsequently, they were baked for 45 min at 200 °C.

### 2.5. Effect of Sourdough on Dough and Bread Properties

The influence of sourdough on the properties of the dough was performed at time zero (T0), after fermentation, and on baked bread. The parameters evaluated in the dough were pH, titratable acidity, and volume increase. In addition, pH, titratable acidity, specific volume, humidity, water activity (a_w_), and color were studied in baked bread.

#### 2.5.1. pH and Titratable Acidity

For the measurement of pH, a G-PH7V-3 VIO solid pH meter with XS 201TN electrode from Labprocess (Badalona, Spain) was used. The method described in Gantumur et al. [11] was followed to measure the titratable acidity. The pH meter used was the VIOLAB version 50 from XS Instruments (Carpi, Italy) and the dispersion device was the T 18 digital ULTRA-TURRAX from the IKA brand (Staufen, Germany). The result was measured in mL of 0.1 M NaOH to reach a pH of 8.5.

#### 2.5.2. Volume Increase

To measure the volume increase of dough fermentation, 5 g of dough were weighed at T0 and pressed onto the bottom of a vial of 50 mL. After fermentation, the growth was measured by a ruler and the result was expressed in cm of dough grown. For this measure, the height of the pre- and post-fermentation dough in the vial was marked with a marker.

#### 2.5.3. Specific Volume

The method explained in Matos and Rosell [12] was followed to calculate the specific volume of bread. The results were expressed in milliliters occupied per gram of sample (mL/g).

#### 2.5.4. Humidity

For the study of bread humidity, crucibles were used in which 10 g of sample were added. The percentage of weight lost was measured after 24 h of samples in the oven at 105 °C. The formula used was:

$$Humidity\;percentage=\left(\frac{\left(M2-M0\right)}{\left(M1-M0\right)}\right)\times 100,$$

where *M*0: weight in g of crucible, *M*1: weight in g of crucible and sample before drying, and *M*2: weight in g of crucible and sample after drying.

#### 2.5.5. Color

To study the color of the bread crust, the CIELAB parameters (L*, a*, b*) were used, as in Galvao et al. [13]. The color of each loaf was measured at three different points on the crust using a Minolta CR-300 colorimeter (Osaka, Japan).

#### 2.5.6. a_w_

Finally, a Humimeter RH2 m (Max-Schaller-Straße, Austria) was used to study the a_w_ of the bread.

### 2.6. Microbiological Analysis

For the microbiological analysis, a count of fungi, yeast, and bacteria was performed in the dough at T0, after fermentation, and in baked bread. To achieve this, 10 g of the sample was introduced into stomacher bags and 90 mL of peptone water (0.1%) was added. Afterward, the mixture was homogenized for 30 s in a Stomacher 400 laboratory mixer (Sewad, AK, USA), obtaining a 1/10 dilution. Subsequently, a serial dilution was made and 100 µL was seeded with a Digralsky loop in plates of two different culture media. The plates with the MRS-A culture medium were incubated in anaerobic jars at 37 °C to isolate the LAB population present [14]. On the other hand, those from PDA were incubated at 28 °C for the isolation of fungi and yeasts, as described by Kazan and Gardiner [15]. All of the plates remained in incubation for 72 h. A triplicates of each sample was made.

### 2.7. Analysis of Antioxidant Activity

DPPH capacity was determined using the method of Zhang et al. [16]. The DPPH (2,2-Diphenyl-1-picrylhydrazyl) radical analysis was performed by adding 400 µL of DPPH solution to 200 µL of a sample. The sample was prepared by adding 30 mL of deionized water to 5 g of sample (dough at T0, fermented dough, and bread). The ULTRA-TURRAX was used for this. The dilutions were then made to obtain absorbances below 1 to establish a linear relationship with the percentage of antioxidant activity. After 60 min of reaction in the dark, absorbance was measured at 517 nm. The results are expressed as a percentage of antioxidant activity (P) using the equation “P (%) = ((C − M)/C) × 100”, in which P is a percentage of antioxidant activity, C is the absorbance of the control, and M is the absorbance of the sample. The blank used was methanol. A triplicate of each sample was made.

### 2.8. Analysis of Total Phenolic Content

The total phenolic content was evaluated using the Folin–Ciocalteu method described by Aryal et al. [17], with minor changes. Then, 130 µL of the sample, 780 µL of deionized water, and 130 µL of Folin–Ciocalteu reagent were used. After mixing, 130 µL of NaCO_3_ was added. The process was performed in the dark for 60 min after being agitated. Finally, the absorbance was measured at 750 nm. The results were measured in milligrams of gallic acid equivalents per kilogram of material (mg GAE/kg). The concentration was calculated using the linear regression equation of the gallic acid calibration curve at values ranging from 0 to 140 mg/L. A triplicate of each sample was made.

### 2.9. Analysis of Organic Acids

For the determination of acetic acid and lactic acid in the samples, the method described in Dopazo et al. [18] was used, adapting it to the case. The samples were diluted 1/20 with deionized water and filtered through a 0.22 μm pore size cellulose filter membrane before analysis. The high-performance liquid chromatography (HPLC) was equipped with a Jasco PU-4180 pump (Easton, MD, USA), a Jasco MD-4015 diode array detector (Easton, MD, USA), a Rezex ROA-Organic Acid (15.097.8 mm) reverse phase column (Phenomenex Inc., Torrance, CA, USA), and a 20 μL sample injection loop. The mobile phase was an isocratic solution of water and SA 0.005 M, flowing at 0.8 mL/min for 10 min and at a temperature of 40 °C. The detector was set at a wavelength of 214 nm for the quantification. A triplicate of each sample was made. Calibration curves were made using lactic acid and acetic acid standards from 0 to 1000 mg/L concentrations. The results were expressed in g/L. The software used for data acquisition and analysis was ChromNAV 2.0 HPLC (Jasco, MD, USA).

### 2.10. Analysis of Volatile Organic Compounds

The determination of volatile organic compounds (VOC) was performed by solid phase headspace microextraction (HS-SPME) and subsequent analysis by gas chromatography coupled with mass spectrometry (GC-MS), following the methodology described by Luz et al. [19], with modifications.

The VOCs of the bread doughs at T0, after fermentation, and once baked were determined. For this, 5 g of sample were homogenized with 20 mL of H_2_O Milli-Q in ULTRA-TURRAX for 3 min. Next, 10 mL of extract was introduced into a 20 mL vial. Headspace VOCs were extracted using a DVB/C-WR/PDMS-coated SPME fiber (80 µm × 10 mm) from Agilent Technologies (Santa Clara, CA, USA). The samples were incubated for 45 min at 50 °C in a water bath with constant agitation.

VOC analysis was performed on an Agilent 7890A gas chromatograph coupled to an Agilent 7000A triple quadrupole mass spectrometer equipped with an electron impact (EI) source. The desorption was performed at 250 °C for 10 min and the injection was conducted in splitless mode. The column used for the chromatographic separation was an HP-5MS (30 m × 0.25 mm, 0.25 µm). The temperature ramp was programmed as follows: 40 °C maintained for 2 min and increased to 160 °C at 6 °C/min, then increased to 260 °C at 10 °C/min and held for 4 min. The carrier gas was helium (99.99%) at a flow rate of 2.5 mL/min. The detection of the compounds was performed in Full Scan mode in an m/z range of 40–450 Da.

The compounds were identified by comparing their mass spectra with those registered in the Mass Spectrometry Data Center (NIST) 09 library. In addition, the linear retention indices (LRI) were calculated based on the retention time of an alkane solution (C8–C20) tested under the same conditions as the samples and compared with the existing literature.

### 2.11. Analysis by Sodium Dodecyl Sulfate-Polyacrylamide Gel Electrophoresis of Bread and Dough

Protein fractions were studied by performing sodium dodecyl sulfate-polyacrylamide gel electrophoresis (SDS-PAGE), according to Ito et al. [20], with minor modifications. First, the doughs at T0, after fermentation, and the baked bread were centrifuged in Eppendorf at 10,822× *g* for 15 min, recovering the supernatants. They were then diluted in a ratio of 1/5 in acetone, mixed in a vortex, and incubated at −20 °C for 30 min. Afterward, they were centrifuged again for 15 min at 17,530× *g*, discarding the supernatant. The protein pellet was resuspended in Milli-Q water using an ultrasonic bath from VWR (Radnor, PA, USA) for 30 min. The samples were mixed in a 1/1 ratio sample buffer pH 6.8 (SDS 2%, glycerol 20%, bromophenol blue 0.01%, and DDT 1.6%) and incubated at 95 °C for 5 min in a VWR thermoblock (Radnor, PA, USA).

Electrophoresis was then performed with a gel from Bio-Rad Laboratories (Hercules, CA, USA) and running buffer (0.3 g of Trizma^®^ Buffer solution, 14.4 g of glycine, and 1 g of SDS per L of Milli-Q water). Each gel well was filled with 20 µL of samples, with 10 µL of a Precision Plus Protein Dual Xtra Standards protein standard (Bio-Rad Laboratories, Hercules, CA, USA) also used as a range protein marker. Conditions were set at 80 V for 30 min and then 120 V for 60 min. Subsequently, the gel was cleaned for 1 min with Milli-Q water, then immersed in Fixing solution (40% MeOH, 10% AcOH, and 50% Milli-Q water) for 30 min under agitation. It was then immersed in staining gel (40% MeOH, 10% AcOH, 50% Milli-Q water) and Comassie R250 0.1%. Finally, the gel was shaken for 24 h in a destaining solution (20% MeOH, 10% AcOH, 70% Milli-Q water). After cleaning the gel with Milli-Q water, the band pattern of the protein compounds was observed.

### 2.12. Biopreservation of Breads

The capacity of conventional sourdough and 5L1 fermented sourdough to avoid contamination by fungal spores in food matrices was evaluated, adapting the methodology described in Tzortzakis [21]. Each type of bread was cut into slices and inoculated by *A. flavus*, *P. verrucosum,* and *P. commune*. The concentration in bread remained at 10^4^ spores/g. For this, a spore suspension was prepared using a Neubauer chamber and four spots of 10 µL were seeded in each slice. Samples were stored in plastic bags and incubated at 25 °C for 7–10 days. The plastic bags containing the slices were examined daily during incubation to determine visible fungal growth and evaluate the shelf life. The shelf life was closed when the bread showed visible signs of fungal growth.

### 2.13. Determination of the Fungal Population on Breads

The fungal population was determined upon completion of the shelf-life study, as previously described in Section 2.6.

### 2.14. Analysis of Mycotoxins in Bread Inoculated by Fungi

The mycotoxin content of the different types of bread inoculated by *A. flavus* and *P. verrucosum* was extracted with the methodology used by Nazareth et al. [22], with a series of modifications. Once the growth of the fungus was apparent in most of the bread, they were weighed in 5 g, placed in a Falcon tube with 25 mL of MeOH, and homogenized for 5 min using an Ultra IKA T 18 basic ULTRA-TURRAX (Staufen, Germany). Following this, the sample was left stirring for 24 h, after which it was centrifuged at 7700× *g*, at 4 °C for 10 min. The supernatant was evaporated to dryness with Büchi Rotavapor R-200 (Postfach, Switzerland). Finally, the dried extracts were resuspended in 1 mL of methanol, filtered with 0.22 µm filters, and vitalized before the analysis with UHPLC (1290 Infinity LC, Agilent Technologies) coupled to a Q-TOF (Agilent 6540 LC/QTOF) mass spectrometer.

The chromatographic separation of the mycotoxins was realized in an Agilent Zorbax RRHD SB-C18 (2.1 × 50 mm, 1.8 µm) column. The mobile phase employed was composed by Milli-Q water 0.1% FA (Phase A) and ACN 0.1% FA (Phase B). The gradient used was configured as: 0 min: 2% B; 22 min: 95% B; 25 min: 5% B. The column was then equilibrated 3 min before the next injection. The flow rate was established at 0.4 mL/min, and the injection volume was 5 µL. For q-TOF analysis, an Agilent Dual Jet Stream electrospray ionization (ESI) was operating in positive ionization mode. The conditions of ESI were configured as follows: gas temperature: 325 °C; gas flow: 10 L/min; nebulizer pressure: 40 psig; sheath gas temperature: 295 °C; sheath gas flow: 12 L/min; capillary voltage: 4000 V; nozzle voltage: 500 V; skimmer: 70 V; scan range: 100–1500 Da; collision energy: 10, 20, 40 eV. For quantification, aflatoxin and ochratoxin calibration curves were prepared, with concentration ranging from 0.01 to 10 mg/L. The integration and data elaboration were realized using MassHunter Qualitative Analysis Software B.08.00.

### 2.15. Statistical Analysis

The statistical analysis of data was performed using Infostat version 2008 software. The experiments were realized in triplicates, with the difference between the control and treated groups analyzed by one-way ANOVA, followed by a Tukey HSD post hoc test for multiple comparisons. The level of significance was considered as *p* ≤ 0.01.

## 3. Results and Discussion

### 3.1. Effect of Sourdough on the Properties of Dough and Bread

The results concerning the influence of sourdough on the properties of dough at different steps of manufacture (T0, after fermentation, and in bread) are plotted in Table 3. The doughs at T0 presented significant differences in pH. SB_2_ had the lowest pH, followed by SB_1_. In contrast, the pH in SBC and SBC_p_ was significantly higher than that of the control bread. The most acidic dough was the one that acted as a control, followed by those added with *L. plantarum* 5L1, with SB_2_ being the most acidic of the two. The acidity of SBC and SBC_p_ was the lowest among the samples.

It was possible to observe that *L. plantarum* 5L1 used in the sourdoughs influenced the pH and titratable acidity prior to dough fermentation. In addition, commercial yeast also had a significant impact on these values. Spontaneous fermentation sourdoughs had less influence on these parameters and, therefore, a slower development. Studies such as Yang et al. [23] also show a higher acidity of sourdough with starter cultures compared to traditional sourdough, being important in the amount of inoculum performed.

After fermentation, pH was reduced in all samples because of the production of acids in the metabolism of yeasts and bacteria. In addition, significant differences were observed between the pH of the different doughs, SB1 and SB2, which had a lower value. The regulation for sourdough bread, which states that the pH after fermentation must be less than 4.8, according to Royal Decree 308/2019 of 26 April 2019 [24], was respected in all situations. Nevertheless, the SBC and SBC_p_ treatments had a higher pH than the control dough.

The titratable acidity increased in all the samples during the fermentation except in the control. In any case, a significantly lower acidity was observed in the doughs with sourdough inoculum compared to the control dough. It seems that the presence of microorganisms directly influences the titratable acidity content.

In studies such as Arendt et al. [25], a drop in pH in sourdough bread was observed. Low pH can have positive effects on the dough, such as increasing the activity of proteases and amylases in the flour and improving the digestibility of bread. In contrast, in this study, the dough inoculated with spontaneous fermentation sourdough did not have a greater pH reduction than the control group. This could happen because the sourdough fermentation lasted only 12 h and did not allow time for the complete development of microorganisms.

Regarding the volume increase results, only a reduced growth rate of the treatment SBC_P_ was detected compared to the control dough. However, it must be considered that the dough fermentation inoculated by sourdough lasted 24 h and that of the dough with commercial yeast only 1 h. In studies such as Sanz-Penella et al. [26], a lower increase in volume is described after fermentation in the sample with 20% sourdough inoculum. In contrast, in this study, the sourdough inoculum had results similar to the control, showing no significant differences. In any case, the amount of sourdough inoculum and the time of fermentation directly influence the dough volume.

The pH of the samples increased after baking. There were significant differences among the values of the loaves of different treatments; however, the pH value coincided with those obtained at T0, comparing the same treatment. The lowest pH occurred in the loaves of bread to which LAB was added, from the highest (treatment S_2_) to the lowest amount of LAB added (treatment S_1_). Thus, only treatment S_2_ complied with the sourdough bread regulations. On the other hand, the control bread had a lower pH than SBC and SBC_p_. Therefore, these results suggested that adding LAB influences the pH of bread even after baking and, in addition, it seemed that the spontaneous fermentation of sourdough might not be enough to generate the expected final pH.

Regarding the titratable acidity, there were also significant differences among the loaves of bread; in particular, the control group showed the highest acidity among treatments. Likewise, treatment S_2_ presented a higher acidity among sourdough bread; SBC and SBC_p_, which had no LAB addition into sourdough, obtained lower titration acidity values. In a general way, the values of titration acidity decreased after baking with respect to the fermented dough step, but the opposite occurred in the control. This may be due to the fact that after the hour of fermentation in the control bread, the yeast population tends to stabilize since, in the fresh yeast formulation, there is a very high concentration of these. This explains the drop in acidity. On the other hand, after baking, the acidity increases again, which indicates that the heat does not greatly affect the acidity in this type of bread. In contrast, in the sourdough inoculum to the dough at T0, the population of bacteria and yeasts in it was still very low compared to commercial fresh yeast. On the other hand, in sourdough bread, there is a drop in acidity after baking, which indicates that heat affects the total acids produced in fermentation. Studies such as Zhang et al. [27] explain how baking bread at high temperatures reduces the viability of the bacteria population in the bread, which may explain this increase in pH and the decrease in acidity.

The specific volume results were significantly higher in SB_1_ and SB_2_ compared to the other samples. This indicates that the crumbs of these breads had a higher porosity. Studies such as Naji-Tabasi et al. [28] showed similar results with a fermentation period longer than 24 h. This effect is due to the breakdown of non-gluten proteins and starch components by sourdough LAB, which becomes more evident in longer fermentation periods [29]. No significant differences between the bread studied were observed regarding a_w_ and humidity. Studies such as Abellana et al. [30] mentioned the importance of these last two parameters in the growth of fungus of the genus *Penicillium* in bakery products.

**Figure 2:**
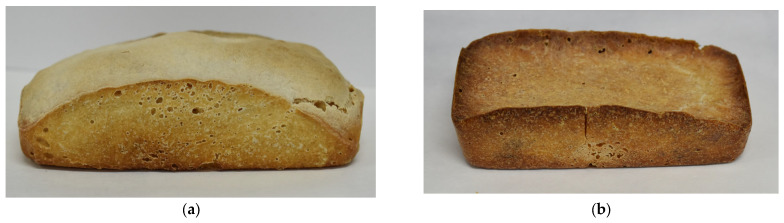

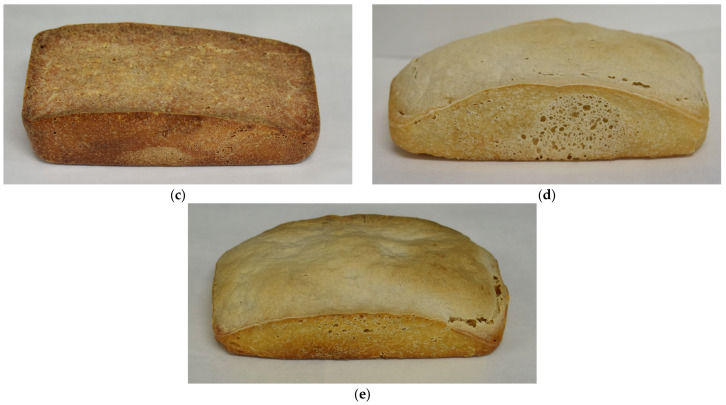
Images of the breads after baking. (**a**): bread control; (**b**): sourdough bread control; (**c**): sourdough bread control with propionate; (**d**): sourdough bread with *L. plantarum* 5L1 inoculum (0.5%); (**e**): sourdough bread with *L. plantarum 5L1* inoculum (5%).

Regarding the color of the bread, significant differences were observed in the lightness parameter (L*) between certain samples, with the control bread being the lightest and SBC_p_ the darkest. The a* parameter, which measures the red/green coordinates, also showed significant differences between the breads studied, with the control bread being the most greenish and the SBC the reddish. Although all of them were close to neutrality between these colors, the control was the only one closest to green. On the other hand, the parameter b*, which measures the yellow/blue coordinates, did not show significant differences between the different breads. Despite this, the integration of *L. plantarum* 5L1 as an ingredient does not visually affect the appearance of the bread, as seen in Figure 2. Therefore, it would not be a problem for the consumer. In studies such as Smith et al. [31], a low-cost method was created to reduce these differences in bread color and thus avoid bias in sensory tests. In our studies, the use of sourdough modified L* and a* parameters regardless of the addition or not of LAB.

### 3.2. Results of Microbiological Analysis

The results of the microbiological population of doughs and breads are observed in Table 4. Regarding the LAB in the doughs at T0, SBC showed the lowest content. In contrast, SBC_p_ had the highest LAB content. There were no significant differences among the rest. These results suggest that propionate avoided the growth of microorganisms, allowing the growth of LAB in general. Therefore, although the association of *L. plantarum* 5L1 and propionate has not been studied, based on results, the addition of propionate could potentialize the growth of *L. plantarum* 5L1 and, consequently, the protective characteristics of this bacteria.

After fermentation, there was a general increase in the viability of the bacteria. Thus, a significantly higher concentration was seen in SBC_p_, SB_1,_ and SB_2_. The concentration was lower in the control dough and in SBC. These results can be explained by the pH at T0, which allowed LAB growth. Regarding the bread, no colonies were detected due to the aggressive heat treatment during baking.

According to the LAB population, the yeast content at T0 was higher in SBC_p_ and SB_2_ and lower in SBC. There was no significant difference between the control and SB_1_. As expected, there was also a general increase in the yeast population in the doughs after fermentation, as with LAB. The highest content was shown by SBC, with a significant difference compared to the control, which had the second-highest concentration. In turn, the content of the control was significantly higher than SB_1_ and SB_2_, between which there was no difference. Finally, the treatment that showed the lowest concentration was SBC_p_.

SBC_p_ was the only treatment in which the yeast and LAB content was reduced after dough fermentation. This was probably due to the antimicrobial action of the propionate. In addition, the theoretical concentration of LAB present in S_1_ and S_2_ was reduced at T0 compared to the concentration of the inoculum. In SB_1_, the concentration of LAB in the form of CFU/g of the sample was reduced in the order of 2 logarithms. In SB_2_, the concentration was reduced in the order of 3 logarithms. These doughs theoretically had LAB concentrations of 10 and 9, respectively, expressed in log_10_ CFU/g of bread once they were inoculated by sourdough. This drop is because the LAB population tends to regulate itself and maintain a sustainable equilibrium. In addition, environmental factors, such as the type of flour, and technological factors, such as aerating the dough, also influence the final ecology of the food [32]. On the other hand, other studies, including Ataç et al. [33], also showed an increase in LAB viability in sourdough made with kefir and flour, with the difference being that they present a longer fermentation of 4 days, compared to 12 h of fermentation for this sourdough. However, in this case, it cannot be seen if there was a reduction in the population when inoculating the sourdough in a dough, since no studies were conducted in bread.

### 3.3. Analysis of Antioxidant Activity

In the results obtained from relative antioxidant activity (Table 4), no significant differences were observed between the doughs before fermenting. After the fermentation, a higher antioxidant activity was seen in SB_1_ and a lower activity in SBC_p_. Finally, the breads did not present significant differences either. In addition, a trend could be observed in which the antioxidant activity decreased after dough fermentation and increased considerably after baking. Studies such as Banu et al. [34] show an increase in antioxidant capacity after sourdough fermentation due to increased levels of phenolic compounds. In this case, however, there were no differences between the breads fermented with a sourdough inoculum and those fermented with commercial yeast.

### 3.4. Analysis of Total Phenolic Content

The results of total phenolic content showed higher amounts of phenolic compounds in the control dough compared to those added from sourdough before fermentation (Table 4). After fermentation, no significant differences were observed between the samples. In the breads, it was possible to appreciate a higher content of phenolic compounds in SB_2_ compared to SBC. On the other hand, there was a general increase in the phenolic content in the samples after fermentation, except for the control dough. In addition, the values decreased after baking, except in the case of SB_2_.

Studies such as Fekri et al. [35] show that the total phenolic content does not vary in the bread with respect to the dough, which means that the benefits of the phenolic compounds last after baking. In this case, the values decreased in the bread compared to the fermented doughs. The only bread in which the total phenolic content increased was SB_2_. This could have led to a significant increase in these values after dough fermentation and the fact that the highest values were maintained after baking, compared to the rest of the breads.

### 3.5. Analysis of Organic Acids

The lactic acid results (Table 4) showed significant differences between the samples in the three times studied. Thus, in the doughs before fermentation, SB_2_ showed the highest value of lactic acid, followed by SB_1_. With lower values than these treatments, the control dough remained and, finally, both doughs were added with conventional sourdough (SBC and SBC_p_). After fermentation, the lactic acid content increased in general in the samples, except for the control dough, which obtained the lowest value of the samples. SBC_p_ had the highest lactic acid content, followed by SB_2_. Finally, in the breads, there were significant differences between all the samples. The one with the highest lactic acid content was SB_1_, followed by SBC_p_ and SB_2_. The control bread showed a higher concentration than SBC.

These results indicate that the addition of lyophilized *L. plantarum* 5L1 as a starter culture in the sourdough resulted in higher production of lactic acid than the spontaneous sourdough. Moreover, adding commercial yeast in the control dough also gives rise to a higher lactic acid content than the spontaneous sourdough. This can be seen in the results at T0. In contrast, after fermentation, the dough inoculated by sourdough showed a higher potential for lactic acid production compared to the control. In the breads, the difference in values was maintained, except in SBC_p_, in which the lactic acid content reduced, reaching values below the control.

Although acetic acid had been evaluated, no production was detected by the different doughs and breads, regardless of the time point analyzed. As indicated by Reis et al. [36], the production of organic acids prolongs the shelf life of food. In particular, the production of lactic acid, because it is related to the acidification of the cytoplasm of the fungal cells, gives rise to a failure in the proton pump. Thus, the highest production of lactic acid by sourdough breads is one of the main reasons for their increased shelf life because it creates an unfavorable environment for the growth of pathogenic fungi [37].

### 3.6. Analysis of Volatile Organic Compounds

The VOC profile varies mainly due to compounds produced during fermentation, enzymatic reactions, lipid oxidation, and thermal shock, according to Wu et al. [38]. The three main pathways for VOC formation are fermentation, in which mainly acids, alcohols, esters, and ketones are produced; lipid oxidation, in which mainly ketones and aldehydes are produced; and the Maillard reaction, which gives rise to pyrazines, pyridines, and pyrroles [39]. These compounds are widely studied in sourdough breads to find new possibilities to improve odor and flavor [38]. A total of 56 compounds were identified in this study and all volatile compounds are described in Table S1.

**Figure 3:**
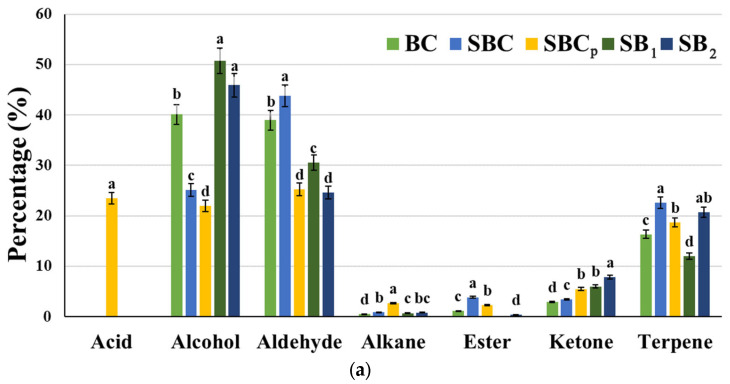

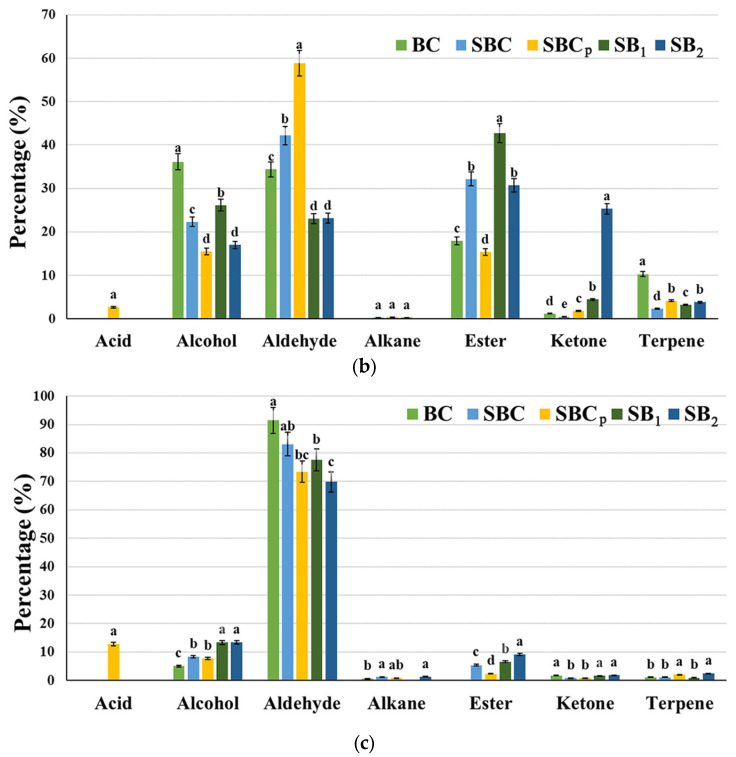
The relative percentage of volatile organic compounds. (**a**): doughs at T0; (**b**): fermented doughs; (**c**): baked breads. BC: bread control; SBC: sourdough bread control; SBC_p_: sourdough bread control with propionate; SB_1_: sourdough bread with *L. plantarum* 5L1 inoculum (0.5%); SB_2_: sourdough bread with *L. plantarum 5L1* inoculum (5%). Different lower-case letters indicate a significant difference in compound concentrations between treatments (*p ≥* 0.05).

A total of 42 VOCs were identified in the doughs at T0 (Figure 3a), including acids (2), alcohols (8), aldehydes (15), alkanes (3), esters (5), ketones (7), and terpenes (2). At a general level, the major compounds were alcohols and aldehydes. The relative percentage of alcohols varied between 22% and 50.8% of the total compounds detected, depending on the sample. In addition, there was a higher concentration in SB_1_ and SB_2_. It is remarkable that the presence of 1-butanol and 3-methyl exists in all the samples except in SBC. The relative percent area of all volatile compounds identified at T0 is described in Table S2. This compound is produced in the metabolism of leucine and phenylalanine by yeasts, according to Wu et al. [38]. In addition, a significant presence of 1-octen-3-ol was also observed in SB_1_ and SB_2_. These two compounds were also detected in sourdough fermented by *L. plantarum* PON100274 for 8 h [40]. In addition, as Pico et al. [41] demonstrated, 1-butanol is described as a compound that positively influences the aroma, while 3-methyl has a negative impact.

Regarding aldehydes, they varied between 24.6% and 39% of the total number of compounds identified. These compounds begin to be produced during kneading, when the fatty acids in the flour bind with oxygen, according to Wu et al. [38]. It is worth noting the presence of hexanal in SB_1_, that of 2-nonenal in dough control, and that of decanal in SBC. In Fang et al. [42], the presence of hexanal was also observed in the sourdough fermented by *L. plantarum*. These compounds are the majority in wheat flour [41]. On the other hand, a considerable presence of terpenes was also seen, varying between 12% and 22.6% depending on the dough. The presence of d-limonene in SBC and the presence of linalool in SBC, SBC_p,_ and SB_2_ is also noteworthy. Regarding acids, they were only detected in SBC_p,_ and both the amount of propionic acid and hexanoic acid was considerable, a consequence of the addition of propionate.

In the doughs after fermentation (Figure 3b), a total of 49 VOCs were identified, among which were acids (1), alcohols (9), aldehydes (16), alkanes (1), esters (15), ketones (5), and terpenes (2). In general, the major compounds were alcohols, aldehydes, and esters. The latter increased during fermentation, due to lipid metabolism and yeast growth, according to Loviso and Libkind [43]. In addition, there was also an increase in the percentage of ketones in SB_2_, an overall decrease in the concentration of terpenes, and a reduction in acids in SBC_p_. The relative percentage of alcohols with respect to the rest of the compounds varied between 15.54% and 36.15%, being the highest in dough control. The relative percent area of all volatile compounds identified in fermented doughs is described in Table S3. The presence of 1-butanol, 3-methyl in all the samples except in SBC_p_ was remarkable. The relative percentage of aldehydes varied between 23.09% and 58.84%, with the significant compounds within this group being hexanal in SBC_p_, 2-heptenal in SB_1_ and SB_2_, and 2-nonenal in SBC. The relative percentage of esters varied between 15.31% and 42.76% and was higher in SBC, SB_1_, and SB_2_. Within this group, the most prominent compound was octanoic acid, ethyl ester, in the doughs mentioned. According to Wu et al. [38], large concentrations of esters can begin to be detected only after 12 h of fermentation in sourdough. Therefore, in the sourdough studied, there was not enough fermentation time to produce compounds of this group, but there was in the 24 h fermentation of the doughs. Within the ketone group, there was an increase after fermentation in SBC_p_ because of the increase in 3-octanone. Liu et al. [44] suggested that this may be due to the fact that LAB fermentation leads to a higher production of ketones. Therefore, SBC_p_, which presents a higher LAB content, showed a higher production of this group of compounds.

A total of 37 VOCs were identified in the baked breads (Figure 3c), including acids (2), alcohols (6), aldehydes (16), alkanes (2), esters (6), ketones (3), and terpenes (2). The relative percent area of all volatile compounds identified in baked breads is described in Table S4. Usually, a 50% reduction in VOC content occurs during baking because many compounds volatilize [38]. The presence of aldehydes was mainly found, with their relative percentage between 69.81% and 91.49%. Within this group, the most prominent compounds were hexanal in all breads and 2-nonenal, which positively influenced the crust of sourdough breads [42]. Pico et al. [41] also showed that aldehydes are the main VOCs in baked bread. Moreover, lipid oxidation reactions produce them in the crumb and are later transferred to the crust. The relative percentage of the rest of the groups decreased drastically. Therefore, to a lesser extent, the presence of alcohol in SB_1_ and SB_2_ stands out due to the content of 1-butanol, 3-methyl. This may be due to the fact that the organic acids produced by *L. plantarum* lead to proteolysis of the flour protein, releasing amino acids, which are aromatic precursors [38]. Within the esters group, they were not detected in the control bread but in the sourdough bread. Furthermore, those containing *L. plantarum* 5L1 showed a higher relative percentage. These compounds are related to the fruity odors of strawberries, pineapple, and bananas [45]. In Hansen and Hansen [46], a higher amount of alcohol and esters was also produced in sourdough bread fermented by a starter culture. In the case of acids, the propionic acid content in SBC_p_ is notable due to the addition of propionate.

### 3.7. Analysis by Sodium Dodecyl Sulfate-Polyacrylamide Gel Electrophoresis of Bread and Dough

**Figure 4:**
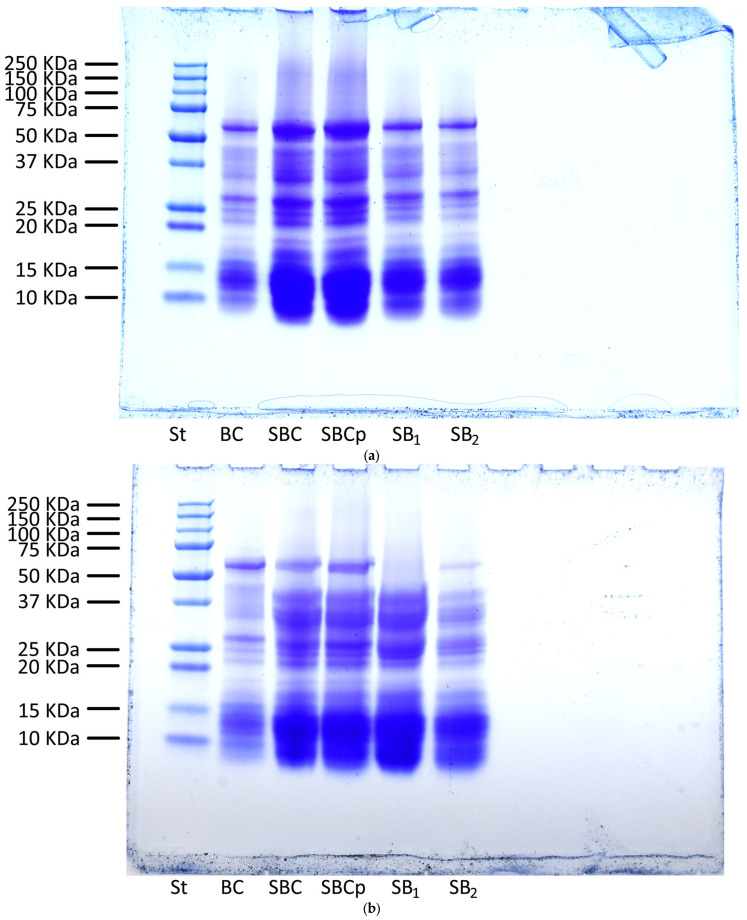

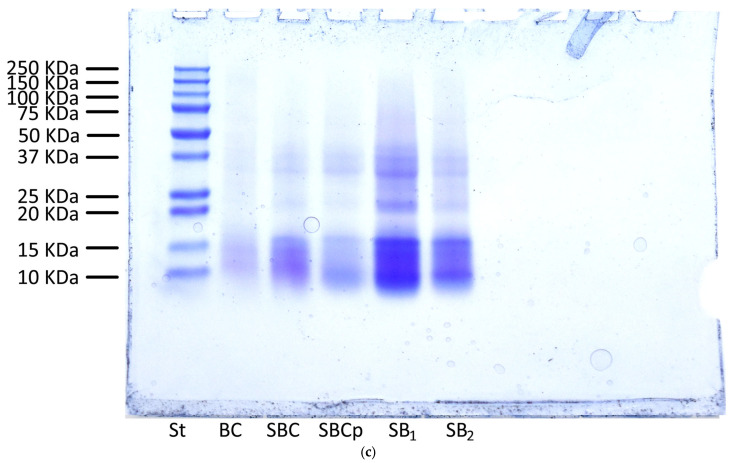
Result of the band pattern of the protein compounds in gel (SDS-PAGE) by electrophoresis. (**a**): doughs at T0; (**b**): fermented doughs; (**c**): baked breads. St: standard; BC: bread control; SBC: sourdough bread control; SBC_p_: sourdough bread control with propionate; SB_1_: sourdough bread with *L. plantarum* 5L1 inoculum (0.5%); SB_2_: sourdough bread with *L. plantarum 5L1* inoculum (5%).

Gluten protein components present in doughs and breads were separated by SDS-PAGE electrophoresis (Figure 4). Among these proteins are gliadins (α, β, γ, and ω); glutenins, separated into high and low MW glutenins; and non-gluten proteins, such as albumin and globulin. Non-gluten proteins are the bands found in the area of molecular weight between 10 and 20 kilo-Daltons (kDa). Gliadins (α, β, γ) are found in the area of molecular weight between 25 and 50 kDa and ω between 50 and 75 kDa. On the other hand, low MW glutenins coincide with the area of glutenins. In contrast, high MW glutenins have an MW greater than 75 kDa. The MW marker used in this study ranged from 10 kDa to 250 kDa, while Abdelaleem and Al-Azab, [47] used a range of 20 kDa to 245 kDa.

The most interesting result in this test was the complete hydrolysis of the proteins found in the 50 kDa band in the dough SB_1_ after fermentation and a partial degradation in the dough SB_2_. These proteins may correspond by MW pattern to the α-gliadins mentioned in De Santis et al. [48], which may be toxic to celiac patients. Therefore, the addition of lyophilized *L. plantarum* 5L1 to the doughs could be interesting to a certain degree to avoid problems in celiac patients.

In contrast, protein hydrolysis did not occur during fermentation in the control and conventional sourdough. Thus, di Cagno et al. [49] showed no differences in the prolamin profile after fermentation in doughs associated with commercial yeast. Moreover, it should be noted that this hydrolysis depends not only on the action of the LAB, but also on an adequate fermentation time, which in this case was 24 h, as evidenced by Diowksz et al. [50].

### 3.8. Biopreservation of Breads

The shelf life results in the loaves showed a similar trend against the three fungi inoculated. In all of them, sourdough bread with propionate prevented the fungus’s visible growth during all the study days. In addition, loaves of sourdough bread had a longer shelf life than control breads, especially the treatment SB_2_ (Table 5).

The loaves of bread inoculated by *A. flavus* had the shortest shelf life because, in the control bread, the visible growth of the fungus began on day 2, and on the third day, all the spots had grown. In SBC and in SB_1,_ the fungal growth began to be visible on the third day, so the shelf life was prolonged compared to the control. In addition, the total growth of the spots occurred on the seventh day. In SB_2_, the visible growth of the fungus began on the fifth day; however, it did not grow in all the spots on the seventh day. In this case, the study of the shelf life in an observational way ended on the seventh day because of the higher infective characteristic of this fungus.

In the case of loaves inoculated by *P. verrucosum*, the growth of the control began on the fourth day and was complete in all spots on the seventh. On the other hand, the growth of SB_1_ began on the fifth day and completed 100% of the spots on the seventh day. In contrast, in SBC, visible growth began on the seventh day, and when the study was stopped on the tenth day, it had only grown in 56% of the inoculated spots. The bread with the better results against the growth of this fungus, apart from the propionate treatment (SBC_p_), was also SB_2_. This sourdough delayed the fungal growth for eight days, and at the end of the experiment, only 19% of the spots showed contamination.

The shelf life was also studied for ten days in the loaves of bread inoculated by *P. commune*. In this case, the fungus in the control group began to grow on the fourth day, similar to the conventional sourdough bread (SBC) and the addition of 1 g of LAB (SB_1_). Despite this, there was a notable difference in the speed of growth. In the control group, the growth of the spots was complete on the fourth day, while in the SBC and SB_1_, it took five days. The S_2_ treatment evidenced fungal growth after the sixth day; the percentage of grown spots increased by 31% on the tenth day.

In studies such as Saladino et al. [51], the antifungal capacity of different LABs against the growth of *A. parasiticus* in sliced breads was evaluated. In this case, no lyophilized LAB was added to the meal preparation, but a cell-free supernatant (CFS) of BAL-fermented media was added. Furthermore, they were added directly into the bread dough and not onto the sourdough. Thus, an increase in the shelf life was observed in breads treated with *L. plantarum* and *L. johnsoni.* In similar studies such as Luz et al. [52], different sourdoughs inoculated by *L. plantarum* and *L. bulgaricus* were fermented to be subsequently lyophilized and used as a powdered ingredient in the preparation of breads. These were inoculated by *P. expansum,* and it was possible to observe an increase in the shelf life of both types of bread with respect to the control. Sourdough with *L. bulgaricus* was added and used as an ingredient; this treatment showed similar shelf-life extension results as the propionate-treated control bread.

### 3.9. Determination of the Fungal Population on Breads

**Figure 5:**
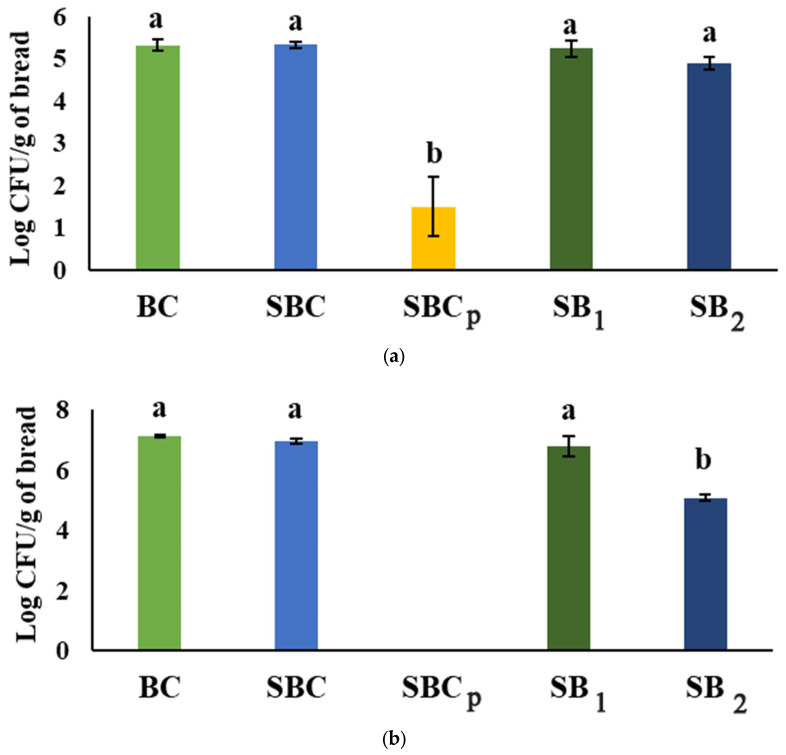

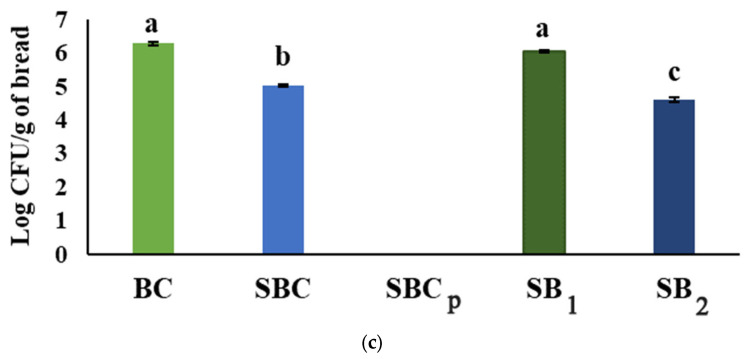
Fungal population in log CFU/g of bread. (**a**): *A. flavus*; (**b**): *P. commune*; (**c**): *P. verrucosum*. BC: bread control; SBC: sourdough bread control; SBC_p_: sourdough bread control with propionate; SB_1_: sourdough bread with *L. plantarum* 5L1 inoculum (0.5%); SB_2_: sourdough bread with *L. plantarum 5L1* inoculum (5%). Different lower-case letters indicate significant differences between treatments at the same time (*p ≥* 0.05).

The results of fungal population in the loaves of bread inoculated by *A. flavus*, *P. commune*, and *P. verrucosum* were obtained in line with the study of the shelf life (Figure 5). In the case of the breads inoculated by *A. flavus*, the treatment in SBC_p_ avoided the fungal growth significantly in comparison to other treatments. In the rest of the treatments, no significant difference was observed after seven days of incubation. Similarly, the addition of propionate in the treatment of SBC_p_ reduced CFU/g in the order of more than 4 logarithms in the fungal population of *P. commune* and *P. verrucosum*. In addition, S_2_ treatment significantly reduced the *P. commune* and *P. verrucosum* growth compared to treatment S_1_ and other groups. In the case of *P. commune*, there were no significant differences between the rest of the breads studied; in those inoculated with *P. verrucosum*, SBC had a lower concentration of spores than the control and SB_1_. In the case of breads inoculated by *Penicillium*, the count was conducted after the tenth day of the observational study of the shelf life. In a general way, the antifungal capacity was proportional to the addition of *L. plantarum*. Studies such as that of Luz et al. [53] also showed a reduction in the fungal population on breads treated with propionate compared to the control, in addition to the potential of sweet whey hydrolyzed in reducing fungal spores when replacing the water in the preparation of bread. Other studies, such as Belz et al. [54], also indicated the potential to reduce fungal growth of propionate and sourdough fermented by *L. amylovorus* DSM 19280 compared to the control bread. In Axel et al. [55], a gluten-free sourdough was also fermented with *L. amylovorus* DSM 19280, improving the shelf life and impacting fungal growth. Although the bacteria were not added to the sourdough in lyophilized form, but in the form of cell suspension, our results corroborated with those exhibited by previous studies, because the addition of LAB may extend the shelf life by preventing fungal growth.

### 3.10. Analysis of Mycotoxins in Bread Inoculated by Fungi

**Figure 6:**
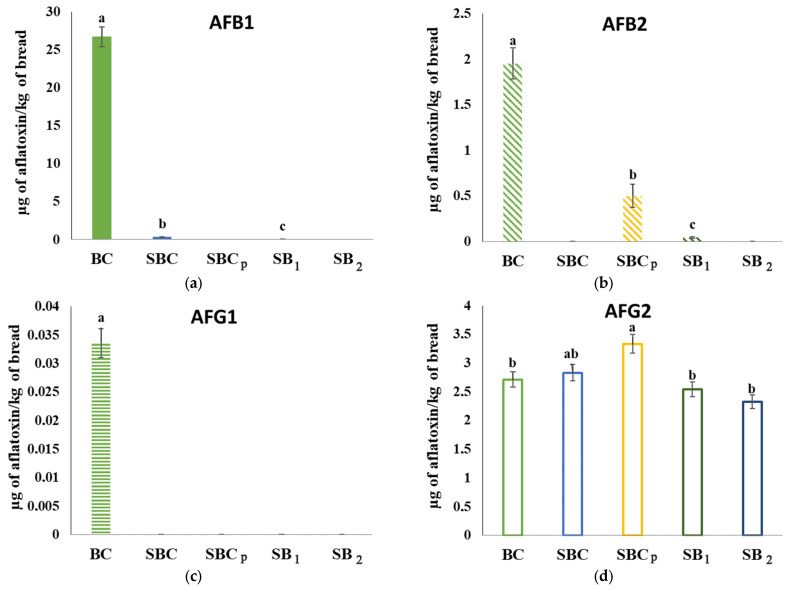
Production of aflatoxins in breads inoculated with *A. flavus*. (**a**): AFB1; (**b**): AFB2; (**c**): AFG1; (**d**): AFG2. The samples received the following treatments: BC: bread control; SBC: sourdough bread control; SBC_p_: sourdough bread control with propionate; SB_1_: sourdough bread with *L. plantarum* 5L1 inoculum (0.5%); SB_2_: sourdough bread with *L. plantarum 5L1* inoculum (5%); AFB1: Aflatoxin B1; AFB2: Aflatoxin B2; AFG1: Aflatoxin G1; AFG2: Aflatoxin G2. Different lower-case letters indicate significant differences in the concentration of a mycotoxin between treatments (*p ≥* 0.05).

In the analysis of the mycotoxin content in breads (Figure 6), the presence of AF was detected in the breads inoculated by *A. flavus*; instead, there was no detection of OTA in the breads inoculated by *P. verrucosum*. In Quiles et al. [56], different cereals were contaminated by these fungi and a greater effect of the application of Allyl Isothiocyanate on the production of OTA was observed than on the production of aflatoxins by these fungi. On the other hand, it is worth adding a higher inhibitory action on fungal growth of the conventional sourdough than that added by *L. plantarum* 5L1 against *P. verrucosum*, further hindering the production of OTA. In addition, *P. verrucosum* is also a producer of AFs, but they were not able to produce AF in this case. In the case of *P. commune*, this analysis was not conducted, since it is not a fungus that produces mycotoxins.

Regarding the breads inoculated by *A. flavus*, the content of Aflatoxin B1 (AFB1), Aflatoxin B2 (AFB2), Aflatoxin G1 (AFG1), and Aflatoxin G2 (AFG2) was analyzed. Thus, the AFB1 content was much higher in the control bread than in the rest, being 26.75 μg/kg of bread. The AFB1 content in the sourdough breads was not significantly different and, in any case, reached 0.5 μg/kg of bread. The same occurred with the AFB2 content, which was significantly higher in the control than in the rest. In this case, on the other hand, the concentration in the control bread was 1.96 μg/kg of bread; in the rest, the highest was 0.5 μg/kg in the treatment SBC_p_. However, there were also no significant differences between sourdough breads. Regarding the AFG1 content, no significant differences were observed between the different breads. Finally, the highest AFG2 content occurred in SBC_p_, having a concentration of 3.34 μg/kg of bread. This presented a significantly higher content than the control bread and the sourdough breads added by *L. plantarum* 5L1. However, the difference was not significantly different from SBC. In the case of AFG2, all the values were approximately 3.34 μg/kg in SBC_p_ and 2.33 μg/kg in SB_2_.

These results evidenced a positive effect of the sourdough inoculum in the breads on the reduction of the content of AFB1 and AFB2 with respect to breads made with commercial yeast. However, no significant differences were observed between the control sourdough loaves and those containing a lyophilized LAB inoculum. In the AFG1 content, no significant differences could be found between any of the breads and, in the case of AFG2, the content was, on the contrary, higher in SBC_p_.

Studies such as Sadeghi et al. [57] showed the antitoxigenic capacity of *L. reuteri* when used as a starter culture in sourdough compared to spontaneous sourdough. In this study, however, there were no significant differences in the use of *L. plantarum* 5L1 as a starter culture to reduce the AF content compared to the spontaneous sourdough. However, a reduction of AFB1 and AFB2 was evidenced in the sourdough breads compared to the control bread. Other studies, such as Illueca et al. [58], showed a reduction in AFB1 production when inoculating the corn surface with *L. plantarum* CECET 749. Thus, the antitoxigenic potential of this LAB in different food matrices is demonstrated. In our study, the addition of *L. plantarum* resulted in antifungal activity which probably led to a reduction in mycotoxin production.

## 4. Conclusions

The studies on bread’s properties showed significant differences in some parameters in sourdough breads added by *L. plantarum 5L1*. Above all, the lowest pH, the highest titratable acidity, and the lowest specific volume occurred in SB_2_. After fermentation, a higher total phenolic content and a higher lactic acid concentration were observed in this bread. Regarding the VOCs of the breads, there was a significant reduction during baking, with aldehydes being the main compounds. To a lesser extent, the presence of alcohols and esters in SB_1_ and SB_2_ stood out. The addition of *L. plantarum* 5L1 in the sourdoughs caused a hydrolysis after fermentation of the proteins found in the 50 kDa band in the dough, corresponding to the MW with the α-gliadins. On the other hand, SB_2_ had a higher antifungal potential. Thus, the growth of *A. flavus* was delayed by three days, that of *P. verrucosum* was delayed by 4 days, and that of *P. commune* was delayed by 2 days, compared to the control.

The results of the mycotoxin content showed the presence of AF in the breads contaminated by *A. flavus*. The sourdough inoculum in the breads produced a reduction in the content of AFB1 and AFB2 compared to the control breads, but there were no differences between the control sourdough breads and those with *L. plantarum* 5L1.

## Figures and Tables

**Table 1:** Sourdough ingredients per 100 g.

Sourdough	Ingredients (g)		
	Wheat Flour	Mineral Water	LAB
SC	33.50	66.50	0.00
S_1_	33.00	66.50	0.50
S_2_	28.50	66.50	5.00

LAB: lactic acid bacteria; SC: sourdough control; S_1_: sourdough with 0.5% lyophilized *L. plantarum* 5L1 inoculated; S_2_: sourdough with 5% lyophilized *L. plantarum* 5L1 inoculated.

**Table 2:** Formulation of breads in g of ingredient per 100 g of product.

Ingredients (g)	BC	SBC	SBC_p_	SB_1_	SB_2_
Wheat flour	60.00	54.25	54.2	54.25	54.25
Mineral water	32.99	17.32	17.1	17.32	17.32
Baker’s yeast	3.70	0.12	0.12	0.12	0.12
Salt	1.31	1.31	1.31	1.31	1.31
Sugar	2.00	2.00	2.00	2.00	2.00
Calcium propionate	0.00	0.00	0.30	0.00	0.00
Sourdough	0.00	25.00	25.00	25.00	25.00

BC: bread control; SBC: sourdough bread control; SBC_p_: sourdough bread control with propionate; SB_1_: sourdough bread with *L. plantarum* 5L1 inoculum (0.5%); SB_2_: sourdough bread with *L. plantarum 5L1* inoculum (5%).

**Table 3:** Results of properties evaluated in doughs before fermentation (T0), after dough fermentation, and after baked breads.

Properties	Dough T0				
	BC	SBC	SBC_p_	SB_1_	SB_2_
(a) pH	5.83 ± 0.04 ^c^	6.02 ± 0.10 ^b^	6.16 ± 0.09 ^a^	5.05 ± 0.02 ^d^	4.77 ± 0.08 ^e^
(b) Titratable acidity (mL NaOH)	14.40 ± 0.36 ^a^	2.50 ± 0.10 ^d^	3.00 ± 0.10 ^d^	4.13 ± 0.06 ^c^	6.33 ± 0.47 ^b^
Properties	Fermented dough				
	BC	SBC	SBC_p_	SB_1_	SB_2_
(a) pH	5.40 ± 0.02 ^a^	4.30 ± 0.62 ^c^	5.13 ± 0.62 ^b^	3.69 ± 0.02 ^d^	3.68 ± 0.45 ^d^
(b) Titratable acidity (mL NaOH)	11.17 ± 0.40 ^a^	9.27 ± 0.85 ^b^	9.57 ± 0.15 ^b^	8.07 ± 0.06 ^b^	8.73 ± 0.31 ^b^
(c) Volume increase (cm)	1.40 ± 0.10 ^a^	1.23 ± 0.06 ^ab^	0.97 ± 0.15 ^b^	0.97 ± 0.06 ^b^	1.07 ± 0.15 ^ab^
Properties	Bread				
	BC	SBC	SBC_p_	SB_1_	SB_2_
(a) pH	5.83 ± 0.04 ^c^	6.02 ± 0.10 ^b^	6.16 ± 0.09 ^a^	5.05 ± 0.02 ^b^	4.77 ± 0.08 ^e^
(b) Titratable acidity (mL NaOH)	14.40 ± 0.36 ^a^	2.50 ± 0.10 ^d^	3.00 ± 0.10 ^d^	4.13 ± 0.06 ^c^	6.33 ± 0.47 ^b^
(c) Specific volume (mL/g)	2.20 ± 0.10 ^a^	2.10 ± 0.10 ^a^	2.20 ± 0.10 ^a^	2.50 ± 0.10 ^b^	2.60 ± 0.11 ^b^
(d) a_w_	0.67 ± 0.05 ^a^	0.68 ± 0.03 ^a^	0.69 ± 0.01 ^a^	0.70 ± 0.03 ^a^	0.72 ± 0.01 ^a^
(e) Humidity (%)	22.30 ± 2.70 ^a^	17.40 ± 0.20 ^a^	18.20 ± 1.70 ^a^	17.40 ± 0.80 ^a^	19.80 ± 0.80 ^a^
(f) Color					
L*	85.00 ± 0.40 ^a^	86.30 ± 0.70 ^bc^	86.00 ± 0.40 ^bc^	86.40 ± 0.20 ^c^	85.40 ± 0.30 ^ab^
a*	0.50 ± 0.20 ^a^	−0.60 ± 0.01 ^c^	−0.10 ± 0.30 ^b^	−0.50 ± 0.20 ^bc^	−0.10 ± 0.20 ^b^
b*	1.50 ± 0.40 ^a^	1.50 ± 0.50 ^a^	1.80 ± 0.30 ^a^	1.70 ± 0.40 ^a^	1.50 ± 0.20 ^a^

BC: bread control; SBC: sourdough bread control; SBC_p_: sourdough bread control with propionate; SB_1_: sourdough bread with *L. plantarum* 5L1 inoculum (0.5%); SB_2_: sourdough bread with *L. plantarum* 5L1 inoculum (5%). Triplicates of each sample were made for analysis. Different lower-case letters indicate significant differences between treatments (*p ≥* 0.05).

**Table 4:** Results of microbiological analysis, antioxidant activity, total phenolic content, and organic acids in doughs before fermentation (T0), after dough fermentation, and after baked breads.

Samples	log CFU/g Sample					
	Dough T0		Fermented Dough		Bread	
	LAB	Yeast	LAB	Yeast	LAB	Yeast
BC	7.42 ± 0.07 ^b^	7.41 ± 0.02 ^b^	9.30 ± 0.09 ^a^	9.44 ± 0.01 ^b^	n.d	n.d
SBC	6.42 ± 0.08 ^c^	5.92 ± 0.10 ^c^	9.29 ± 0.23 ^a^	10.81 ± 0.11 ^a^	n.d	n.d
SBC_p_	8.22 ± 0.13 ^a^	8.16 ± 0.03 ^a^	8.00 ± 0.11 ^b^	8.10 ± 0.02 ^d^	n.d	n.d
SB_1_	7.47 ± 0.03 ^b^	7.47 ± 0.03 ^b^	7.95 ± 0.13 ^b^	8.90 ± 0.08 ^c^	n.d	n.d
SB_2_	7.69 ± 0.01 ^b^	8.19 ± 0.09 ^a^	7.80 ± 0.01 ^b^	8.78 ± 0.04 ^c^	n.d	n.d
	Relative antioxidant activity (%)					
	Dough T0		Fermented dough		Bread	
BC	60.07 ± 10.16 ^a^		54.22 ± 9.72 ^ab^		67.63 ± 7.29 ^a^	
SBC	55.00 ± 8.32 ^a^		47.15 ± 7.09 ^ab^		62.96 ± 3.57 ^a^	
SBC_p_	53.19 ± 21.14 ^a^		45.85 ± 0.61 ^b^		54.96 ± 7.55 ^a^	
SB_1_	63.96 ± 0.55 ^a^		65.59 ± 0.39 ^a^		66.22 ± 0.39 ^a^	
SB_2_	51.89 ± 14.38 ^a^		47.63 ± 0.31 ^ab^		67.52 ± 0.71 ^a^	
	Total phenolic content (mg of gallic acid equivalents/kg)					
	Dough T0		Fermented dough		Bread	
BC	93.99 ± 4.27 ^a^		80.66 ± 8.26 ^a^		69.45 ± 2.52 ^ab^	
SBC	72.05 ± 5.10 ^b^		78.96 ± 7.64 ^a^		67.02 ± 8.50 ^b^	
SBC_p_	69.33 ± 2.41 ^b^		92.30 ± 2.12 ^a^		72.05 ± 7.03 ^ab^	
SB_1_	75.27 ± 8.80 ^b^		79.57 ± 7.94 ^a^		76.05 ± 4.64 ^ab^	
SB_2_	72.24 ± 1.11 ^b^		86.84 ± 8.77 ^a^		89.08 ± 5.86 ^a^	
	Lactic acid (g/kg)					
	Dough T0		Fermented dough		Bread	
BC	0.49 ± 0.02 ^c^		0.43 ± 0.01 ^d^		0.76 ± 0.01 ^d^	
SBC	0.36 ± 0.01 ^d^		1.18 ± 0.05 ^c^		0.70 ± 0.03 ^e^	
SBC_p_	0.32 ± 0.01 ^d^		1.66 ± 0.03 ^a^		1.79 ± 0.01 ^b^	
SB_1_	0.58 ± 0.01 ^b^		1.16 ± 0.02 ^c^		2.03 ± 0.02 ^a^	
SB_2_	0.86 ± 0.03 ^a^		1.31 ± 0.01 ^b^		1.27 ± 0.01 ^c^	

BC: bread control; SBC: sourdough bread control; SBC_p_: sourdough bread control with propionate; SB_1_: sourdough bread with *L. plantarum* 5L1 inoculum (0.5%); SB_2_: sourdough bread with *L. plantarum* 5L1 inoculum (5%); n.d: not detected. In all assays, a triplicate of each sample was made for analysis. Different lower-case letters indicate significant differences between treatments at the same time (*p ≥* 0.05).

**Table 5:** Results of the study of the shelf life of the different bread samples contaminated with spores of *A. flavus*, *P. verrucosum*, and *P. commune.* Each bread was cut into four slices with four contamination spots.

Breads	Fungi	Percentage of Grown Spots (Days)									
		1	2	3	4	5	6	7	8	9	10
BC	*A. flavus*	0 ± 0 ^a^	38 ± 14 ^a^	100 ± 0 ^a^	100 ± 0 ^a^	100 ± 0 ^a^	100 ± 0 ^a^	100 ± 0 ^a^	-	-	-
SBC		0 ± 0 ^a^	0 ± 0 ^b^	19 ± 13 ^b^	31 ± 13 ^c^	75 ± 0 ^b^	88 ± 14 ^a^	100 ± 0 ^a^	-	-	-
SBC_p_		0 ± 0 ^a^	0 ± 0 ^b^	0 ± 0 ^c^	0 ± 0 ^d^	0 ± 0 ^d^	0 ± 0 ^c^	0 ± 0 ^c^	-	-	-
SB_1_		0 ± 0 ^a^	0 ± 0 ^b^	38 ± 14 ^b^	69 ± 13 ^b^	94 ± 13 ^a^	94 ± 13 ^a^	100 ± 0 ^a^	-	-	-
SB_2_		0 ± 0 ^a^	0 ± 0 ^b^	0 ± 0 ^c^	0 ± 0 ^d^	19 ± 13 ^c^	56 ± 13 ^b^	75 ± 0 ^b^	-	-	-
BC	*P. verrucosum*	0 ± 0 ^a^	0 ± 0 ^a^	0 ± 0 ^a^	31 ± 13 ^a^	56 ± 13 ^a^	88 ± 14 ^a^	100 ± 0 ^a^	100 ± 0 ^a^	100 ± 0 ^a^	100 ± 0 ^d^
SBC		0 ± 0 ^a^	0 ± 0 ^a^	0 ± 0 ^a^	0 ± 0 ^b^	0 ± 0 ^b^	0 ± 0 ^b^	13 ± 14 ^b^	19 ± 13 ^b^	38 ± 14 ^b^	56 ± 12 ^c^
SBC_p_		0 ± 0 ^a^	0 ± 0 ^a^	0 ± 0 ^a^	0 ± 0 ^b^	0 ± 0 ^b^	0 ± 0 ^b^	0 ± 0 ^b^	0 ± 0 ^c^	0 ± 0 ^c^	0 ± 0 ^a^
SB_1_		0 ± 0 ^a^	0 ± 0 ^a^	0 ± 0 ^a^	0 ± 0 ^b^	63 ± 14 ^a^	94 ± 13 ^a^	100 ± 0 ^a^	100 ± 0 ^a^	100 ± 0 ^a^	100 ± 0 ^d^
SB_2_		0 ± 0 ^a^	0 ± 0 ^a^	0 ± 0 ^a^	0 ± 0 ^b^	0 ± 0 ^b^	0 ± 0 ^b^	0 ± 0 ^b^	6 ± 13 ^bc^	13 ± 14 ^bc^	19 ± 13 ^b^
BC	*P. commune*	0 ± 0 ^a^	0 ± 0 ^a^	94 ± 13 ^a^	100 ± 0 ^a^	100 ± 0 ^a^	100 ± 0 ^a^	100 ± 0 ^a^	100 ± 0 ^a^	100 ± 0 ^a^	100 ± 0 ^a^
SBC		0 ± 0 ^a^	0 ± 0 ^a^	69 ± 13 ^a^	88 ± 14 ^a^	100 ± 0 ^a^	100 ± 0 ^a^	100 ± 0 ^a^	100 ± 0 ^a^	100 ± 0 ^a^	100 ± 0 ^a^
SBC_p_		0 ± 0 ^a^	0 ± 0 ^a^	0 ± 0 ^b^	0 ± 0 ^b^	0 ± 0 ^b^	0 ± 0 ^b^	0 ± 0 ^b^	0 ± 0 ^b^	0 ± 0 ^c^	0 ± 0 ^c^
SB_1_		0 ± 0 ^a^	0 ± 0 ^a^	69 ± 13 ^a^	88 ± 14 ^a^	100 ± 0 ^a^	100 ± 0 ^a^	100 ± 0 ^a^	100 ± 0 ^a^	100 ± 0 ^a^	100 ± 0 ^a^
SB_2_		0 ± 0 ^a^	0 ± 0 ^a^	0 ± 0 ^b^	0 ± 0 ^b^	0 ± 0 ^b^	6 ± 13 ^b^	6 ± 13 ^b^	6 ± 13 ^b^	19 ± 13 ^b^	31 ± 13 ^b^

BC: bread control; SBC: sourdough bread control; SBC_p_: sourdough bread control with propionate; SB_1_: sourdough bread with *L. plantarum* 5L1 inoculum (0.5%); SB_2_: sourdough bread with *L. plantarum* 5L1 inoculum (5%). Different lower-case letters indicate significant differences in fungal growth in different treatments on the same day (*p ≥* 0.05).

## Data Availability

The data presented in this study are available on request from the corresponding author.

## Supplementary Materials

The following supporting information can be downloaded at: https://www.mdpi.com/article/10.3390/foods12040864/s1, Table S1: Results of volatile compounds identified in doughs before fermentation, after fermentation and after baked breads, Table S2. Relative area percentage of volatile organic compounds in doughs at time point zero. (C): control group without sourdough; (SC1): control sourdough with spontaneous fermentation; (SC2): control sourdough with spontaneous fermentation plus propionate; (S1): sourdough group with 0.5% of lyophilized Lactobacillus. plantarum 5L1; and (S2): sourdough group with 5% of lyophilized *L. plantarum* 5L1, Table S3: Relative area percentage of volatile organic compounds in fermented doughs. (C): control group without sourdough; (SC1): control sourdough with spontaneous fermentation; (SC2): control sourdough with spontaneous fermentation plus propionate; (S1): sourdough group with 0.5% of lyophilized Lactobacillus. plantarum 5L1; and (S2): sourdough group with 5% of lyophilized *L. plantarum* 5L1, Table S4: Relative area percentage of volatile organic compounds in breads. (C): control group without sourdough; (SC1): control sourdough with spontaneous fermentation; (SC2): control sourdough with spontaneous fermentation plus propionate; (S1): sourdough group with 0.5% of lyophilized Lactobacillus. plantarum 5L1; and (S2): sourdough group with 5% of lyophilized *L. plantarum* 5L1.
