# Supplementary material for: Bread Biopreservation through the Addition of Lactic Acid Bacteria in Sourdough
Source: Foods. 2023 Feb 17;12(4):864. doi: 10.3390/foods12040864 (PMC9956393; doi:10.3390/foods12040864)
Supplement: Supplementary file 1 [file foods-12-00864-s001.zip › foods-2184171-supplementary.pdf]

Article

Bread biopreservation through the addition of lactic acid bacteria in sourdough

Francisco Illueca [1,†], Ana Moreno [2,†], Jorge Calpe [2], Tiago Melo Nazareth [1,*], Victor D'Opazo [1], Giuseppe Meca [1], Juan Manuel Quiles [1], and Carlos Luz [1]

1 Department of Food Science and Toxicology, Faculty of Pharmacy, University of Valencia, Ave. Vicent Andrés Estellés s/n, 46100, Burjassot, Spain

2 AgrotechUV incubator, Scientific Park of University of Valence, St. Catedrático Agustín Escardino 9, 46980, Paterna, Spain.

* Correspondence: tiago@uv.es; Tel.: +34963544959

† These authors contributed equally to this work and should be considered co-first authors.

Table S1. Results of volatile compounds identified in doughs before fermentation, after fermentation and after baked breads.

Peak	Rt	Compound	Identification	LRI DB5	LRI lit
**Acid**					
1	4.26	Propanoic acid	MS		
2	6.22	Hexanoic acid	MS + LRI	979	978
**Alcohol**					
3	3.41	1-Butanol, 3-methyl	MS		
4	6.18	1-Hexanol	MS + LRI	877	878
5	8.69	1-Heptanol	MS + LRI	980	977
6	8.71	1-Octen-3-ol	MS + LRI	980	978
7	9.14	5-Hepten-2-ol, 6-methyl	MS + LRI	998	994
8	10.05	1-Hexanol, 2 ethyl	MS + LRI	1034	1038
9	11.18	1-Octanol	MS + LRI	1079	1076
10	13.29	3-Nonen-1-ol	MS + LRI	1167	1157
11	13.59	1-Nonanol	MS + LRI	1179	1172
**Aldehyde**					
12	3.97	Hexanal	MS		
13	6.26	Heptanal	MS + LRI	881	882
14	8.23	2-Heptenal	MS + LRI	960	961
15	8.54	Benzaldehyde	MS + LRI	974	970
16	8.84	Octanal	MS + LRI	986	981
17	9.72	2,4-Heptadienal	MS + LRI	1020	1013
18	9.82	5-Ethylcyclopent-1-enecarboxaldehyde	MS + LRI	1025	1026
19	10.89	2-Octenal	MS + LRI	1068	1071
20	11.04	Benzeneacetaldehyde	MS + LRI	1074	1074
21	11.42	Nonanal	MS + LRI	1089	1089
22	13.42	2-Nonenal	MS + LRI	1172	1168
23	13.91	Decanal	MS + LRI	1192	1188

24	15.12	2,4-Nonadienal	MS + LRI	1245	1236
25	15.82	2-Decenal	MS + LRI	1276	1274
26	16.93	2,4-Decadienal	MS + LRI	1326	1325
27	18.12	2-Undecenal	MS + LRI	1381	1373
**Alkane**					
28	9.23	Undecane	MS + LRI	1100	1100
29	14.09	Tridecane	MS + LRI	1300	1300
30	16.36	Tetradecane	MS + LRI	1400	1400
31	22.49	Heptadecane	MS + LRI	1700	1700
**Ester**					
32	3.64	Butanoic acid ethyl ester	MS		
33	5.27	Acetic acid, pentyl ester	MS + LRI	837	859
34	5.77	Pentanoic acid, ethyl ester	MS + LRI	859	871
35	10.62	Isobutyric acid, pentyl ester	MS + LRI	1057	1057
36	10.72	Heptanoic acid, ethyl ester	MS + LRI	1061	1081
37	10.95	Propanoic acid, hexyl ester	MS + LRI	1070	1089
38	11.48	2,4-Hexadienoic acid, ethyl ester	MS + LRI	1091	1093
39	11.52	Benzoic acid, methyl ester	MS + LRI	1092	1094
40	13.03	Isobutyric acid, hexyl ester	MS + LRI	1156	1151
41	13.16	Octanoic acid, ethyl ester	MS + LRI	1161	1173
42	15.48	Nonanoic acid, ethyl ester	MS + LRI	1261	1268
43	15.74	Acetic acid, 2-phenyl ethyl ester	MS + LRI	1272	1264
44	17.69	Decanoic acid, ethyl ester	MS + LRI	1361	1373
45	17.78	Propanoic acid, 2-phenylethyl ester	MS + LRI	1365	1353
46	21.77	Dodecanoic acid, ethyl ester	MS + LRI	1562	1566
47	27.43	Hexadecanoic acid, ethyl ester	MS		
**Ketone**					
48	7.27	2-Heptanone, 4-methyl	MS + LRI	923	936
49	8.32	1-Octen-3-one	MS + LRI	965	962
50	8.43	3-Octanone	MS + LRI	969	971

51	8.6	2,5-Octanedione	MS + LRI	976	983
52	10.38	3-Octen-2-one	MS + LRI	1047	1046
53	11.3	2-Nonanone	MS + LRI	1084	1089
54	16.12	2-Undecanone	MS + LRI	1289	1291
**Terpene**					
55	7.93	D-Limonene	MS + LRI	1049	1039
56	11.57	Linalool	MS + LRI	1095	1098

Table S2. Relative area percentage of volatile organic compounds in doughs at time point zero. (C): control group without sourdough; (SC_1): control sourdough with spontaneous fermentation; (SC_2): control sourdough with spontaneous fermentation plus propionate; (S_1): sourdough group with 0.5% of lyophilized *Lactobacillus. plantarum* 5L1; and (S_2): sourdough group with 5% of lyophilized *L. plantarum* 5L1.

Peak	Compound	C	SC_1	SC_2	S_1	S_2
**Acid**		**n.d**	**n.d**	**23.50**	**n.d**	**n.d**
1	Propanoic acid	n.d	n.d	12.10	n.d	n.d
2	Hexanoic acid	n.d	n.d	11.40	n.d	n.d
**Alcohol**		**40.10**	**25.10**	**22.00**	**50.80**	**45.90**
3	1-Butanol, 3-methyl	12.30	2.90	13.10	13.50	15.50
4	1-Hexanol	9.40	6.30	n.d	24.00	15.50
5	1-Octen-3-ol	8.70	8.10	6.00	10.40	11.60
6	5-Hepten-2-ol, 6-methyl	0.40	0.80	0.40	0.40	0.50
7	1-Hexanol, 2 ethyl	2.40	3.80	0.40	0.50	0.40
8	1-Octanol	1.60	1.30	1.10	1.40	1.40
9	3-Nonen-1-ol	4.10	1.20	0.50	0.30	0.40
10	1-Nonanol	1.20	0.70	0.50	0.30	0.60
**Aldehyde**		**39.00**	**43.80**	**25.20**	**30.60**	**24.60**
11	Hexanal	2.00	6.40	n.d	12.50	3.90
12	Heptanal	n.d	1.40	3.30	n.d	n.d
13	2-Heptenal	5.00	3.10	3.70	5.30	6.50
14	Benzaldehyde	n.d	0.40	n.d	n.d	n.d
15	Octanal	0.50	1.00	0.60	1.10	1.10
16	2,4-Heptadienal	n.d	n.d	n.d	0.30	0.40
17	5-Ethylcyclopent-1-enecarboxaldehyde	1.00	1.70	2.50	1.50	1.80
18	2-Octenal	1.70	2.90	3.30	3.20	3.00
19	Benzeneacetaldehyde	9.20	0.30	0.50	0.60	0.30
20	Nonanal	1.00	2.30	3.60	1.40	2.30
21	2-Nonenal	11.80	3.10	4.90	1.60	2.50

22	Decanal	6.40	20.30	0.90	0.40	0.90
23	2,4-Nonadienal	n.d	n.d	1.10	1.00	0.40
24	2,4-Decadienal	0.50	0.90	0.80	1.30	1.50
25	2-Undecenal	n.d	n.d	n.d	0.40	n.d
**Alkane**		**0.50**	**0.90**	**2.70**	**0.70**	**0.80**
26	Tridecane	n.d	n.d	0.90	0.40	0.40
27	Tetradecane	0.20	0.90	1.60	0.30	0.40
28	Heptadecane	0.30	n.d	0.20	n.d	n.d
**Ester**		**1.10**	**3.80**	**2.30**	**n.d**	**0.40**
29	Isobutyric acid, pentyl ester	n.d	0.60	n.d	n.d	n.d
30	Isobutyric acid, hexyl ester	n.d	2.20	2.30	n.d	n.d
31	Octanoic acid, ethyl ester	0.80	n.d	n.d	n.d	0.40
32	Nonanoic acid, ethyl ester	n.d	1.00	n.d	n.d	n.d
33	Decanoic acid, ethyl ester	0.30	n.d	n.d	n.d	n.d
**Ketone**		**2.90**	**3.40**	**5.50**	**6.00**	**7.80**
34	2-Heptanone, 4-methyl	n.d	0.40	n.d	n.d	0.30
35	1-Octen-3-one	n.d	n.d	2.30	2.60	3.30
36	3-Octanone	1.50	n.d	n.d	1.00	n.d
37	2,5-Octanedione	0.40	1.00	1.30	0.80	0.90
38	3-Octen-2-one	0.40	0.80	1.00	0.90	1.40
39	2-Nonanone	0.60	0.90	0.90	0.70	1.40
40	2-Undecanone	n.d	0.30	n.d	n.d	0.50
**Terpene**		**16.30**	**22.60**	**18.70**	**12.00**	**20.70**
41	D-Limonene	6.80	10.80	7.00	4.40	9.30
42	Linalool	9.50	11.80	11.70	7.60	11.40

Table S3. Relative area percentage of volatile organic compounds in fermented doughs. (C): control group without sourdough; (SC_1): control sourdough with spontaneous fermentation; (SC_2): control sourdough with spontaneous fermentation plus propionate; (S_1): sourdough group with 0.5% of lyophilized *Lactobacillus. plantarum* 5L1; and (S_2): sourdough group with 5% of lyophilized *L. plantarum* 5L1.

Peak	Compound	C	SC_1	SC_2	S_1	S_2
**Acid**		**n.d**	**n.d**	**2.65**	**n.d**	**n.d**
1	Propanoic acid	n.d	n.d	2.65	n.d	n.d
**Alcohol**		**36.15**	**22.31**	**15.54**	**26.16**	**16.99**
3	1-Butanol, 3-methyl	19.84	16.43	4.89	19.8	11.51
4	1-Hexanol	6.94	1.43	3.12	1.14	1.00
5	1-Heptanol	n.d	0.59	n.d	n.d	n.d
6	1-Octen-3-ol	5.36	1.60	4.17	2.44	2.50
7	5-Hepten-2-ol, 6-methyl	0.36	n.d	n.d	n.d	n.d
8	1-Hexanol, 2 ethyl	n.d	0.45	0.44	n.d	n.d
9	1-Octanol	0.75	1.19	1.94	1.19	0.90
10	3-Nonen-1-ol	1.81	0.32	0.50	0.42	0.45
11	1-Nonanol	1.09	0.30	0.48	1.17	0.63
**Aldehyde**		**34.38**	**42.2**	**58.84**	**23.09**	**23.19**
12	Hexanal	2.23	4.85	20.39	3.91	1.38
13	Heptanal	n.d	0.44	n.d	0.91	n.d
14	2-Heptenal	9.50	9.32	8.26	12.75	12.38
15	Benzaldehyde	0.44	n.d	0.26	n.d	n.d
16	Octanal	0.43	0.19	0.95	0.41	0.35
17	2,4-Heptadienal	n.d	n.d	0.26	n.d	n.d
18	5-Ethylcyclopent-1-enecarboxaldehyde	0.92	0.74	1.94	0.23	0.26
19	2-Octenal	1.44	2.44	6.44	1.45	1.12
20	Benzeneacetaldehyde	3.00	0.20	0.22	0.21	0.25
21	Nonanal	0.78	0.83	1.55	0.83	0.61

22	2-Nonenal	9.81	18.98	6.62	n.d	5.37
23	Decanal	4.83	2.65	5.88	0.48	0.34
24	2,4-Nonadienal	0.43	0.55	1.87	0.72	0.37
25	2-Decenal	n.d	n.d	0.74	0.46	0.25
26	2,4-Decadienal	0.57	0.76	2.99	0.41	0.51
27	2-Undecenal	n.d	0.25	0.47	0.32	n.d
**Alkane**		**n.d**	**0.27**	**0.44**	**0.31**	**n.d**
30	Tetradecane	n.d	0.27	0.44	0.31	n.d
**Ester**		**17.97**	**32.18**	**15.32**	**42.76**	**30.71**
32	Butanoic acid ethyl ester	0.72	3.73	0.92	0.33	0.25
33	Acetic acid, pentyl ester	n.d	4.03	n.d	8.97	2.40
34	Pentanoic acid, ethyl ester	0.48	n.d	n.d	0.25	n.d
35	Isobutyric acid, pentyl ester	1.66	0.23	0.23	n.d	n.d
36	Heptanoic acid, ethyl ester	0.82	0.92	0.82	1.31	1.41
37	Propanoic acid, hexyl ester	n.d	n.d	0.61	n.d	n.d
38	2,4-Hexadienoic acid, ethyl ester	n.d	n.d	n.d	0.25	3.70
40	Isobutyric acid, hexyl ester	n.d	n.d	0.71	n.d	n.d
41	Octanoic acid, ethyl ester	9.79	14.23	6.61	17.66	17.05
42	Nonanoic acid, ethyl ester	0.35	0.55	1.74	0.79	0.72
43	Acetic acid, 2-phenyl ethyl ester	0.47	1.12	0.43	2.80	0.67
44	Decanoic acid, ethyl ester	3.23	6.67	1.91	9.01	3.73
45	Propanoic acid, 2-phenylethyl ester	n.d	n.d	0.69	n.d	n.d
46	Dodecanoic acid, ethyl ester	0.45	0.32	0.22	0.56	0.30
47	Hexadecanoic acid, ethyl ester	n.d	0.38	0.43	0.83	0.48
**Ketone**		**1.21**	**0.47**	**1.83**	**4.44**	**25.29**
50	3-Octanone	n.d	0.24	0.37	3.71	24.3
51	2,5-Octanedione	0.40	n.d	0.57	0.20	0.32
52	3-Octen-2-one	0.40	n.d	0.23	n.d	n.d
53	2-Nonanone	0.41	0.23	0.41	0.32	0.38

54	2-Undecanone	n.d	n.d	0.25	0.21	0.29
**Terpene**		**10.32**	**2.31**	**4.16**	**3.25**	**3.85**
55	D-Limonene	4.66	1.20	2.83	1.69	2.04
56	Linalool	5.66	1.11	1.33	1.56	1.81

Table S4. Relative area percentage of volatile organic compounds in breads. (C): control group without sourdough; (SC_1): control sourdough with spontaneous fermentation; (SC_2): control sourdough with spontaneous fermentation plus propionate; (S_1): sourdough group with 0.5% of lyophilized *Lactobacillus. plantarum* 5L1; and (S_2): sourdough group with 5% of lyophilized *L. plantarum* 5L1.

**Peak**	**Compound**	**C**	**SC_1**	**SC_2**	**S_1**	**S_2**
**Acid**		**n.d**	**n.d**	**12.78**	**n.d**	**n.d**
1	Propanoic acid	n.d	n.d	10.8	n.d	n.d
2	Hexanoic acid	n.d	n.d	1.98	n.d	n.d
**Alcohol**		**5.03**	**8.36**	**7.69**	**13.34**	**13.43**
3	1-Butanol, 3-methyl	1.51	5.44	4.77	9.98	11.11
4	1-Hexanol	0.50	0.61	n.d	0.88	0.53
5	1-Octen-3-ol	1.94	1.37	1.51	1.00	0.98
6	1-Hexanol, 2 ethyl	0.54	0.50	0.78	n.d	n.d
7	1-Octanol	0.54	0.44	0.63	0.61	0.44
8	1-Nonanol	n.d	n.d	n.d	0.87	0.37
**Aldehyde**		**91.49**	**83.07**	**73.41**	**77.53**	**69.81**
9	Hexanal	49.41	35.86	21.21	23.44	35.65
10	Heptanal	2.76	1.60	1.48	1.37	1.17
11	2-Heptenal	3.63	3.20	2.51	3.76	3.01
12	Benzaldehyde	4.26	2.93	3.01	1.20	2.23
13	Octanal	3.24	1.72	0.92	0.96	1.71
14	2,4-Heptadienal	n.d	0.53	n.d	n.d	n.d
15	5-Ethylcyclopent-1-enecarboxaldehyde	2.53	0.96	0.90	0.77	n.d
16	2-Octenal	6.73	4.00	2.20	2.98	2.88
17	Benzeneacetaldehyde	0.52	0.56	6.32	1.50	6.66
18	Nonanal	3.99	3.00	2.17	1.41	1.92
19	2-Nonenal	3.91	21.22	25.22	36.87	10.24
20	Decanal	2.19	3.82	6.05	1.16	2.03
21	2,4-Nonadienal	3.63	1.46	0.90	1.05	1.04

22	2-Decenal	2.29	1.24	0.52	0.69	0.87
23	2,4-Decadienal	0.96	0.52	n.d	n.d	n.d
24	2-Undecenal	1.44	0.45	n.d	0.37	0.40
**Alkane**		**0.62**	**1.21**	**0.87**	**n.d**	**1.31**
25	Undecane	n.d	0.48	n.d	n.d	0.89
26	Tetradecane	0.62	0.73	0.87	n.d	0.42
**Ester**		**n.d**	**5.40**	**2.37**	**6.60**	**9.16**
27	Acetic acid, pentyl ester	n.d	0.48	n.d	0.59	4.53
28	Isobutyric acid, pentyl ester	n.d	n.d	n.d	n.d	0.97
29	2,4-Hexadienoic acid, ethyl ester	n.d	n.d	n.d	n.d	0.45
30	Octanoic acid, ethyl ester	n.d	2.69	1.42	3.33	2.01
31	Acetic acid, 2-phenyl ethyl ester	n.d	0.67	0.45	1.35	0.40
32	Decanoic acid, ethyl ester	n.d	1.56	0.50	1.33	0.80
**Ketone**		**1.75**	**0.81**	**0.83**	**1.57**	**1.87**
33	1-Octen-3-one	1.10	0.81	0.83	1.12	1.38
34	3-Octanone	n.d	n.d	n.d	0.45	0.49
35	3-Octen-2-one	0.65	n.d	n.d	n.d	n.d
**Terpene**		**1.12**	**1.14**	**2.04**	**0.95**	**2.40**
36	D-Limonene	0.59	0.77	1.17	0.53	1.91
37	Linalool	0.53	0.37	0.87	0.42	0.49
